# Postpriapism erectile dysfunction and shunt-related urethral stricture: long-term morbidity after proximal shunt for ischaemic priapism

**DOI:** 10.11604/pamj.2014.19.72.5209

**Published:** 2014-09-24

**Authors:** Jibril Oyekunle Bello

**Affiliations:** 1 Urology unit, Department of Surgery, University of Ilorin Teaching Hospital, Ilorin, Nigeria

**Keywords:** Ischaemic priapism, cavernoso-spongiosal shunt, Postpriapism erectile dysfunction, shunt-related urethral stricture

## Abstract

Recent guidelines have advocated for step-wise treatment of increasing invasiveness in the management of ischaemic priapism though with low-level evidences. In the past, proximal shunts were favoured as first-line treatment. We present an African man who had proximal shunt (cavernoso-spongiosal) three decades ago for ischaemic priapism and subsequently had long-term morbidity over the three decades with adverse effect on his quality of life. Recent guidelines appear to be sound despite their limitations and more invasive cavernoso-spongiosal shunts may be associated with significant long-term morbidities and poor quality of life.

## Introduction

It is only in the last decade or two that evidences and guidelines have advocated for a step-wise pattern of increasing invasiveness in the surgical treatment of acute episodes of ischaemic priapism [1]. This is practiced without compromising the goal of achieving early detumescence and preservation of erectile function. Three decades ago, many of the patients were treated primarily with proximal shunts often with dismissal of the usefulness of corporal aspiration and irrigation and at the time, no knowledge of the effectiveness of distal shunts [2]. Proximal shunts were popular procedures carried out by surgeons during this period and Quackel's cavernoso-spongiosal shunt was often hailed as the procedure of choice for ischaemic priapism [2, 3]. Several reports have described urethro-cutaneous fistulas as a common early complication of the cavernoso-spongiosal shunt [2, 3]. We report a case of an elderly African man who had long-term morbidity over three decades with associated negative effect on his quality of life following cavernoso-spongiosal shunt done for ischaemic priapism during his early adulthood.

## Patient and observation

A 30-year-old African man with sickle cell trait (AS) developed ischaemic priapism in 1981. He had cavernoso-spongiosal shunt with complete detumescence. However, he developed urethro-cutaneous fistula in the early post-operative period which was repaired after six months with Devine-Horton urethroplasty technique. He was subsequently lost to follow-up. Thirty-one years later, he was referred back to the same hospital where he previously had his shunt created by his family physician. The referral followed the finding of a bladder stone in a Pelvic X-ray done when he complained of recurrent pelvic and perineal pains. On further evaluation, he described three decades-long history of bothersome lower urinary tract symptoms (LUTS) and he also had erectile dysfunction(ED) often improvising with digital support for successful vaginal penetration. This resulted in a recurrent acrimonious relationship with his spouse due to unsatisfying sexual intercourse. He was however able to father eight children during this period despite the ED. His International prostate symptom score (IPSS) was 18 and International index of erectile function-5 (IIEF-5) score was 12. Physical examination revealed meatal stenosis, a 5cm ventral midline distal penile scar of prior penile surgery and distal penile urethral induration.

**Figure 1:**
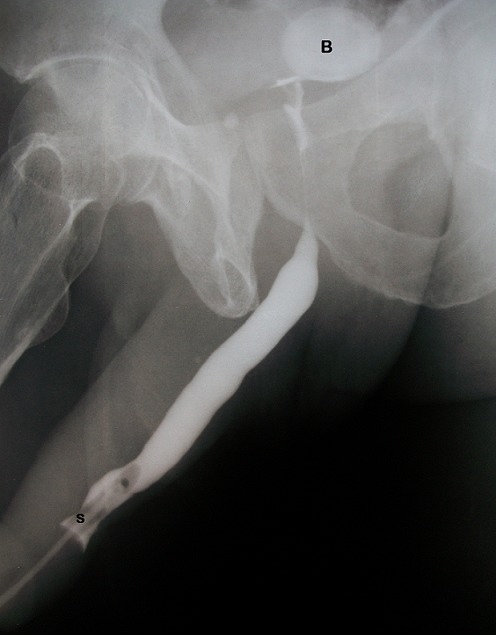
Retrograde urethrogram: illustration of the narrowing and irregularity in the distal penile urethra (S) and Bladder stone (B)

**Figure 2:**
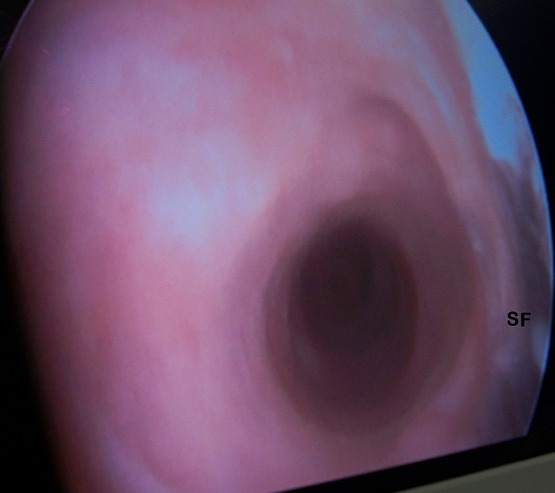
Image at urethrocystoscopy showing the narrowing, scaring and fibrosis at distal urethra in the area of the previously created shunt (SF)

Prostate examination revealed no enlargement. A diagnosis of Postpriapism erectile dysfunction and shunt-related urethral stricture was made. He had abdominopelvic ultrasound done which showed no upper urinary tract abnormality, prostate volume of 30 mLs and a 3.5cm echogenic oval structure in the bladder casting an acoustic shadow. Retrograde urethrogram revealed irregularity and some narrowing in the distal-penile urethra (Figure 1). His Prostate specific antigen was 1.8ng/mL and serum electrolytes were within normal limits. He had urethrocystoscopy done after meatal dilatation and findings were narrowing, scarring and fibrosis in the distal-penile urethra at the site of the previous cavernoso-spongiosal shunt (Figure 2). Urethrocystoscopy also revealed a non-obstructing prostate, two bladder stones, the larger of which was approximately 3.5cm. The distal urethral stricture was easily dilated and cystolapaxy carried out. He declined shunt reversal. He was discharged on the third day and later commenced on oral Phosphodiesterase-5 inhibitors for the ED (Tabs Tadalafil 10mg on-demand). At one-year follow-up the LUTS had improved significantly (IPSS = 7), erectile function also improved to mild (IEFF Score of 20) and he was having more satisfying sexual intercourse than he had in the previous three decades.

## Discussion

Ischaemic priapism is an emergency urologic condition and presents as persistent painful penile erection that continues hours beyond or is unrelated to sexual stimulation [1]. Ischemic priapism is not uncommon in haemoglobinopathies and other haematologic disorders; however, it is rarely reported in those with sickle cell trait [4]. A variety of surgical treatment options are available, all aiming to achieve early detumescence and preservation of erectile function. Recent guidelines have advocated a step-wise treatment of increasing level of invasiveness though this was made with limited supporting evidence due to rarity of the condition and availability of only small studies or case series [1].

In earlier times, proximal shunts were created as first-line treatment often with dismissal of the usefulness of less invasive measures as providing only temporary relief [2]. At the time, the patho-physiology of priapism was poorly understood with no knowledge of the role of vasoconstrictors and distal shunts were yet to be popularized. Cavernoso-spongiosal shunt was a popular surgical procedure for ischaemic priapism three decades ago and several reports have documented urethro-cutaneous fistula as a common complication of the procedure [2, 3]. Some modifications were attempted during this era to prevent this complication with some success [2, 3]. Our patient developed priapism during that era, had cavernoso-spongiosal shunt created and developed urethro-cutaneous fistulae in the immediate post operative period. This was later repaired by Devine-Horton urethroplasty. Our patient's clinical recent re-presentation suggests an early development of shunt-related urethral stricture with subsequent long history of LUTS and the development of bladder stones. Erectile function may be impaired following ischaemic priapism as a result of primary damage from prolonged ischemia and secondary fibrosis; ED could also result from the treatment given due to the creation of shunts. Our patient had mild to moderate ED for the three decades after his shunt was created and this was a significant bother to him and his spouse. Phosphodiesterase-5 inhibitor was effective in improving the postpriapism erectile dysfunction in our patient; others have reported closure of the created shunt as treatment for postpriapism ED [5]. Our patient declined shunt closure as he wanted no further open penile surgeries; he reported an improvement in his quality of life with better voiding and improved erectile function at one-year follow-up.

## Conclusion

Guidelines advocating step-wise treatment of increasing invasiveness for ischaemic priapism appear to be sound despite low-level evidence. The more invasive cavernoso-spongiosal shunts may be associated with morbidities not only in the immediate post operative period but also long-term.
